# Feasibility of home hand rehabilitation using musicglove after chronic spinal cord injury

**DOI:** 10.1038/s41394-022-00552-4

**Published:** 2022-11-09

**Authors:** Quentin Sanders, Vicky Chan, Renee Augsburger, Steven C. Cramer, David J. Reinkensmeyer, Kelli Sharp

**Affiliations:** 1 Department of Bioengineering, George Mason University, Fairfax, VA USA; 2 Department of Mechanical Engineering, George Mason University, Fairfax, VA USA; 3 Rehabilitation Services, University of California Irvine Medical Center, Irvine, CA USA; 4 California Rehabilitation Hospital, Los Angeles, CA USA; 5 Department of Neurology, University of California, Los Angeles, Los Angeles, CA USA; 6 Department of Mechanical & Aerospace Engineering, University of California Irvine, Irvine, CA USA; 7 Department of Biomedical Engineering, University of California Irvine, Irvine, CA USA; 8 Department of Physical Medicine and Rehabilitation, University of California Irvine, Irvine, CA USA; 9 Department of Anatomy and Neurobiology, University of California, Los Angeles, CA USA; 10 Department of Dance, University of California Irvine, Irvine, CA USA

**Keywords:** Spinal cord, Randomized controlled trials

## Abstract

**Study design:**

Randomized, controlled single-blind cross over study. This study was registered on ClinicalTrials.gov (NCT02473614).

**Objectives:**

Examine usership patterns and feasibility of MusicGlove for at home hand rehabilitation therapy following chronic spinal cord injury.

**Setting:**

Homes of participants.

**Methods:**

Ten participants with chronic spinal cord injury completed two baseline assessments of hand function. After a stable baseline was determined all participants were randomized into two groups: Experimental and Control. Each group was given a recommended therapy dosage. Following this participants switched interventions.

**Results:**

On average participants had higher levels of compliance (6.1 ± 3.5 h.), and completed more grips (15,760 ± 9,590 grips) compared to participants in previous stroke studies using the same device. Participants modulated game parameters in a manner consistent with optimal challenge principles from motor learning theory. Participants in the experimental group increased their prehension ability (1 ± 1.4 MusicGlove, 0.2 ± 0.5 Control) and performance (1.4 ± 2.2 MusicGlove, 0.4 ± 0.55 Control) on the Graded and Redefined Assessment of Strength, Sensibility, and Prehension subtests. Increases in performance on the Box and Blocks Test also favored the experimental group compared to the conventional group at the end of therapy (4.2 ± 5.9, −1.0 ± 3.4 respectively).

**Conclusions:**

MusicGlove is a feasible option for hand therapy in the home-setting for individuals with chronic SCI. Participants completed nearly twice as many gripping movements compared to individuals from the sub-acute and chronic stroke populations, and a number far greater than the number of movements typically achieved during traditional rehabilitation.

## Introduction

The number of movements achieved in traditional in-clinic therapy sessions after spinal cord injury (SCI) typically are below the number of repetitions thought necessary to help facilitate cortical reorganization [1]. Home based rehabilitation programs have been prescribed as an approach to increase the number of repetitions achieved outside of the clinic. These programs often consist of following a printed-out hand out of exercises prescribed by a therapist, but compliance with performing a list of hand exercises prescribed for home-based rehabilitation therapy is often poor [2]. Low levels of compliance often can arise from several factors. For instance, people may not initiate the recommended treatment or they may prematurely discontinue therapy. One key factor often linked to compliance is the level of motivation of the individual, with high compliance to a protocol typically indicating an increased level of motivation to perform the therapy [2].

Although they are different patient populations, in the stroke rehabilitation literature there have been many studies that have shown that the use of wearable movement sensors coupled with computer games can be motivating for rehabilitation [3–8]. For example, in our lab, we previously developed the MusicGlove a type of wearable movement sensor. The glove is instrumented with sensors on each of the fingertips and lateral aspect of the index finger [4, 9]. Prior studies performed in our lab found that MusicGlove motivated individuals with chronic stroke to perform hundreds of functional gripping movements during 30-min training sessions and that exercise with the device led to significantly greater improvements in hand grasping ability than conventional hand training [4]. In a home-based training study after chronic stroke, users who used the MusicGlove reported greater improvements in self-reported functional use of their impaired limb compared to those who followed a book of hand exercises, suggesting that MusicGlove was feasible and effective for home hand rehabilitation [5]. Additionally, in a home-based training study in the subacute phase after stroke, we found that the MusicGlove motivated individuals to perform more gripping movements in comparison to the number completed by those in the chronic study, while choosing game difficulty settings that allowed them to achieve high levels of note hitting success at the game [10].

While there have been moderate levels of success with wearable movement sensing approaches (such as MusicGlove) for hand rehabilitation in the stroke population demonstrated by our lab and others [4, 5, 8, 10], there have been very few studies that have attempted to apply the same technology to hand rehabilitation in persons with SCI. One study examined the use of a wearable glove with integrated vibration motors for hand rehabilitation after chronic SCI [11]. The glove was designed to teach piano melodies passively and small DC motors in the glove stimulated the fingers during the day in the order of the notes in the songs to be learned. The study was conducted with 10 participants with incomplete spinal cord injuries in C4 – T1 and showed improvements in sensation measured via Semmes-Weinstein’s test at the end of the study. However, no improvements in motor function where observed in the study. Further, this approach differs from the wearable movement sensing approach as these approaches typically do not provide haptic stimulation or assistance but merely detect movement, which is used to control a computer game.

Thus, it remains unclear if wearable movement sensors as previously defined above are a feasible option for hand rehabilitation following SCI. The purpose of the present study thus was to evaluate the feasibility of using a wearable movement sensor for hand rehabilitation after SCI. Specifically, given the results that our lab has shown with the MusicGlove device we sought to investigate the use of MusicGlove in the SCI population for hand rehabilitation after SCI. Our aim in this manuscript is to discuss three feasibility goals and their related outcomes. First, the MusicGlove was originally designed for use by individuals with a stroke, and using it requires a moderate level of preserved hand function in the donning hand to self-don the device at home, and to make the necessary gripping movements to play the game. The first aim of the current study was to compare how users with hand impairment after SCI used the device at home in comparison to previously reported cohorts of stroke survivors (subacute, and chronic) who had used the device at home in previous studies. Also, a common concern about self-administered care in the home setting is whether subjects will appropriately challenge themselves. In the study with subacute stroke patients [10], we found that they challenged themselves in a way consistent with the Challenge Point Hypothesis, which states that training is optimized when difficulty is modulated based on performance. Thus the second aim was to characterize how users with a SCI chose game parameters and to determine if users progressively challenged themselves as they progressed through therapy. Finally, we sought to establish a preliminary estimate of the therapeutic effect of MusicGlove use on hand function in subjects with chronic SCI.

## Methods

### Subjects

The University of California at Irvine Institutional Review Board approved this study, and all participants provided informed consent prior to enrollment in the study. Both the inclusion and exclusion criteria for the study are listed in Table 1. It should be noted that the inclusion criteria provided in table one contain more detail on study implementation than the ones listed on the original clinicaltrials.gov registration. All demographic and baseline clinical data of the participants are shown in Table 2.

**Table 1:** Inclusion and exclusion criteria.

Inclusion criteria	Exclusion criteria
Between the ages of 18 to 80	Severe reduced level of consciousness
History of spinal cord injury affecting hand function at least six months prior to enrollment	Severe sensory/proprioception deficit at the affected upper extremity (score = 0 on the Nottingham sensory assessment)
Able to perform at least 3 blocks on the Box and	Currently pregnant
Block Test (BBT) with the training hand	Difficulty in understanding or complying with instructions given by the experimenter
No other active major neurological disease other than the spinal cord injury	Inability to perform the experimental task that will be studied
Absence of pain in the training upper extremity – score ≤ 3 on the visual analog pain scale	Increased pain with movement of the affected upper extremity

**Table 2:** Demographic & baseline data of subjects.

	MusicGlove group (*n* = 5)	Control group (*n* = 5)
Age (years)	49.4 ± 18.1	53.2 ± 14.8
Time Since Spinal Cord Injury (Months)	54.1 ± 33.9	173.21 ± 149
Gender	4 M/1 F	4 M/1 F
Hand with Greater Impairment	2 R/3 L	2 R/3 L
Box and Blocks Test (BBT)	40.6 ± 1.7	40 ± 18
GRASSP: Strength	29.6 ± 5.6	27.6 ± 5.5
GRASSP: SWMT Dorsal	8.2 ± 3.6	8.4 ± 4.5
GRASSP: SWMT Palmar	8.8 ± 3.7	9.2 ± 3.4
GRASSP: Prehension ability	18.6 ± 3.0	18.6 ± 6.1
GRASSP: Prehension performance	7.0 ± 1.4	7.2 ± 2.2
Modified Ashworth Scale: Elbow	0.60 ± 0.80	0.60 ± 0.80
Modified Ashworth Scale: Wrist	1.00 ± 0.63	0.80 ± 0.75
Modified Ashworth Scale: Fingers	1.60 ± 1.02	1.00 ± 1.10
ASIA Impairment Scale (AIS)	D: 4, C: 1	D: 3, C: 2

### Outcome measures

An experienced, blinded rehabilitation therapist performed a set of clinical assessments at both baseline visits and at each additional time point during the study. The primary feasibility outcome measure was the amount of use of MusicGlove, defined as the number of grasping movements made and the number of hours of use. The Box and Blocks Test (BBT) score and Graded Redefined Assessment of Strength, Sensation and Prehension (GRASSP) test score measured at the end of the first three-week training period (end of training, EOT) were preregistered on clincialtrials.gov as the primary and secondary clinical outcome measures, respectively. The BBT is a standard clinical assessment of hand function that evaluates unilateral gross manual dexterity in an individual by observing how many blocks a subject can pick up, and place from one side of a box to the other in 60 seconds [12]. While the GRASSP test consists of five subtests that are used to assess sensorimotor hand function in persons with chronic spinal cord injuries that are representative of three domains vital to hand function: strength, sensibility, and prehension [13].

### Interventions

Subjects received two types of interventions in this study: a booklet of conventional hand therapy exercises and the MusicGlove device. The exercise booklet consisted of 18 different standard exercises for home training of the hand and were developed by an experienced occupational therapist. For each exercise a total of 2 sets were performed, twice a day with a varying number of repetitions (i.e. 3,4,10, or 12 repetitions) and hold durations (i.e. 1,3,4,5, or 10 seconds) depending on the activity. A detailed list of the exercise can be found in the supplementary material. MusicGlove is a relatively low-cost device (price of MusicGlove $349, price of most robotic hand therapy devices > $5000) instrumented glove with six electrical leads located on all five finger tips and one on the proximal interphalangeal joint on the lateral aspect of the index finger. When the lead on the thumb touches any of the other five leads, an electrical connection is closed which is then registered by the computer as an event. A USB controller was built to interface the glove with a computer. A PIC 18F14K50 microcontroller sends a unique output to a computer corresponding to each of the five digital inputs it receives. In order to operate the MusicGlove users make functional gripping movements by touching the sensor on the tip of the thumb to one of the other five sensors coordinated with scrolling notes that are displayed on a video game screen as music plays. When an event corresponding to a particular grip type is received from the glove, the software checks if there is a corresponding note in the “hit window” on the game (a variable threshold based on song difficulty). If there is a note that is triggered as a “hit” then it is recorded as successful “hit”, but if not the grip event is ignored. In total there were five different types of grips that the user could make, a key-pinch grip and opposition of the thumb to each of the four fingers [10].

### Intervention protocol

**Fig. 1:**
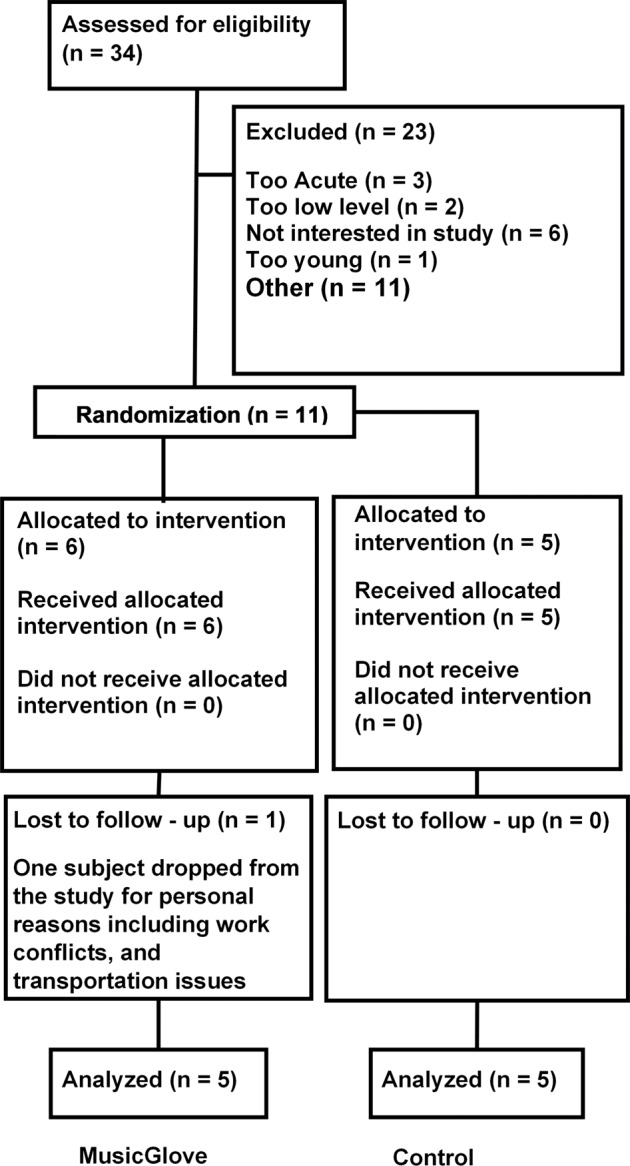
Consort flow diagram. The diagram displays the phases in which the study progresses, beginning with patient screening, followed by randomization, and subsequent analysis.

For all subjects two baseline measures were taken 3–10 days apart for both the BBT as well as the GRASSP test to establish a stable baseline. After the initial baseline assessment, subjects were randomized based on their BBT score and their age into either the MusicGlove group or the control group by alternating block allocation, a technique referred to as adaptive randomization [5] (Fig. 1). Subjects in each group were then instructed on how to engage with their assigned intervention. It should be noted that the rehabilitation therapist was not present when the interventions were given, and was blinded to how participants were distributed between groups. The experimental group (MusicGlove group) began with the MusicGlove device, while the control group was given a booklet of tabletop exercises for home training of the hand developed by experienced occupational therapists. Subjects who received the booklet of hand therapy exercises were given instructions as well as a demonstration on how to correctly perform each exercise in the home setting.

Subjects who received the MusicGlove could modulate the difficulty of their MusicGlove training by changing the number of grip types (1–5: key pinch grip, index-thumb grip, middlethumb grip etc.) needed to play each song, and/or by selecting songs at three different difficulty levels (Easy, Medium, Difficult), where the difficulty was determined by the number of musical notes presented per minute of song. Additionally, MusicGlove group subjects could also play the game in two different modes: song mode and session mode.

In “song” mode subjects were presented with the option to modify the game parameters (song difficulty or number of grips used) after each individual song (~ 3 minutes in duration) or replay the same song with the exact same parameters. While in “session” mode a number of songs were played in series for a set duration (~ 15 minutes). During session mode the song difficulty and number of grips used stayed the same, but a different song was played each time. It should be noted however that after every song participants still had the option of ending “session mode” early and modifying the parameters. Participants were also not instructed to play in one mode over the other, and the option of which mode to play in was left to the participants.

Both groups were instructed to perform self-guided hand training for at least three hours per week spread over at least three sessions per week for three weeks. After three weeks, the subjects returned for post-therapy clinical assessments (end-of-therapy). Subjects were then instructed to cease exercise for three weeks, and then return at a follow-up one month after the post-therapy assessment for their first long-term clinical assessment.

After the first long-term assessment was completed, subjects initially in the conventional therapy group were given a MusicGlove while subjects in the experimental group returned their MusicGlove and were now given the conventional hand therapy exercise booklet. Both subjects were asked to repeat the same therapy regiment described earlier (3 h./wk. for 3 wks.). At the end of this crossover exercise period, these subjects returned for a second round of post-therapy assessments and returned the intervention they had been given. Afterwards, they returned one month later for their third follow-up assessment.

### Statistical analysis

All statistical analyses were performed using the MATLAB programming software [14]. We selected a sample size of 10 appropriate for initial pilot testing in the SCI population. To characterize how users with a SCI chose game parameters and to determine if users progressively challenged themselves as they progressed through therapy we analyzed data from periods of use of the MusicGlove device for usership metrics for each subject including: note hitting success (# of notes completed / # of notes presented), amount of practice (as measured by the # of grips presented and the total usage time), and the types of in-game adjustments (i.e. changing song difficulty or grip types used) made. We tested whether the probability of making a parameter change on the next song depended on the level of success achieved with the previous song using linear regression.

Additionally, Spearman’s correlation coefficient test was used to compare usage patterns (usage across each day) in the current study to that seen in previous study with individuals in the sub-acute phase of stroke. Two-sided, paired t-tests were used to compare the level of success at hitting the notes as well as the song difficulty level chosen by the user between the present study, and the previous study with subacute stroke users. These analyses were performed to compare how users with hand impairment after SCI used the device in comparison to previously reported cohorts of stroke survivors (subacute, and chronic) who had used the device in previous studies.

We performed an exploratory analysis of the relationship between demographic data, MusicGlove usership metrics, and individual BBT scores. Specifically, Spearman’s Correlation Coefficient test was performed between the initial baseline BBT scores, age, and the MusicGlove usership metrics with Bonferroni’s correction applied for multiple comparisons. The datasets that were generated during the current study also are available from the corresponding author on reasonable request.

## Results

### Usage patterns: amount of use of MusicGlove

**Fig. 2:**
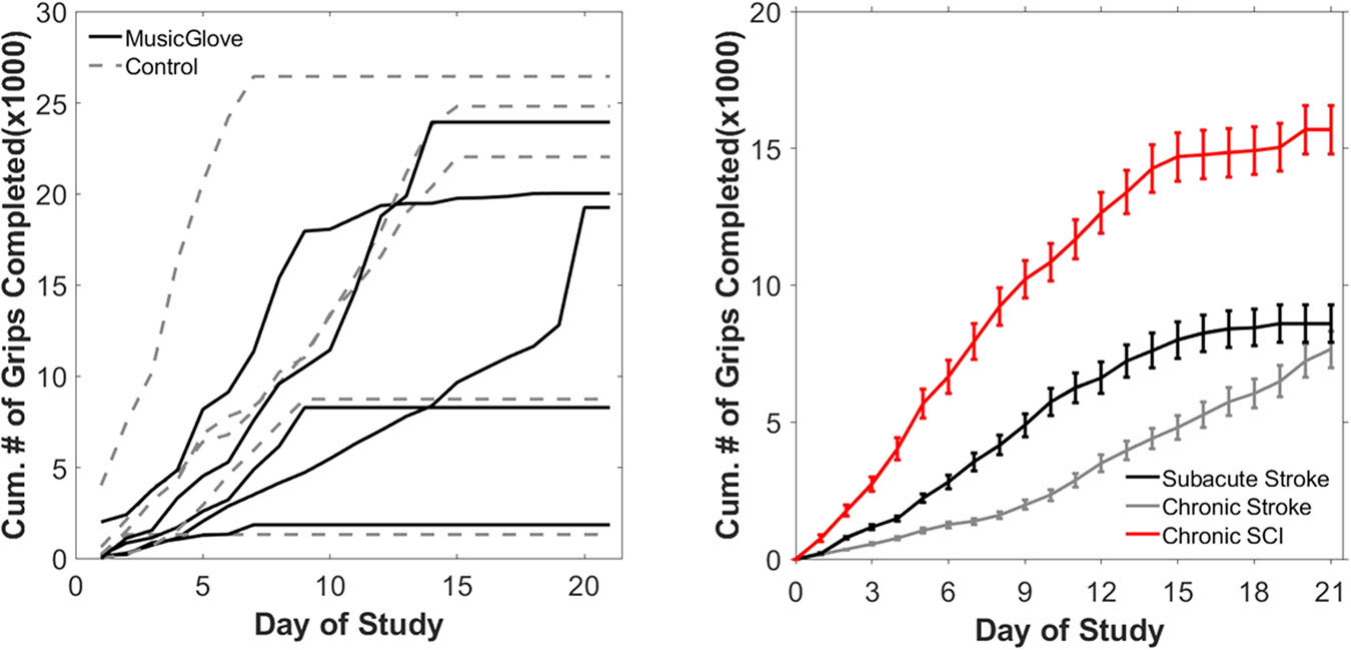
Summary of usership of the MusicGlove device. (Left) The cumulative number of grips completed by each subject in the group that received the MusicGlove first (MusicGlove), and the group that used the MusicGlove second, after three weeks of conventional home therapy (Control). (Right) The average cumulative number of grips completed by the subjects from the current study compared to number completed by chronic and subacute stroke survivors from two previous studies [30]. Bars show ± 1 SE.

The MusicGlove computer data logs revealed that the 10 subjects used the device on average 6.1 ± 3.5 hours, which was 68% of the recommended 9 hours. For comparison, in the previous study conducted with individuals in the subacute phase of stroke subjects used the device 4.1 ± 3.2 hours, which equates to 46% of the recommended therapy time (although this difference was not statistically significant, p = 0.2, two-sided paired t-test). Usage time was unavailable for the study conducted with chronic stroke users preventing the comparison between the present study, and those users. Subjects in the current study completed on average a total of 15,760 ± 9,590 grips, which is nearly double the amount that was completed in the sub-acute and chronic stroke studies (8,627 ± 7,500 grips and 6,953 ± 6,546 grips respectively) (Fig. 2). This difference however, only trended towards significance (p = 0.074 when compared to sub-acute stroke, and p = 0.069 when compared to chronic stroke). Additionally, subjects in the present study significantly decreased the number of grips completed during week 3 compared to week 1 (paired t-test, p = 0.05) (Fig. 2). This trend was also seen in the study with individuals in the sub-acute phase of stroke (rho = 0.9, p < 0.005, Spearman correlation coefficient test), but not in the study with individuals in the chronic phase.

### Usage patterns: challenge selection

**Fig. 3:**
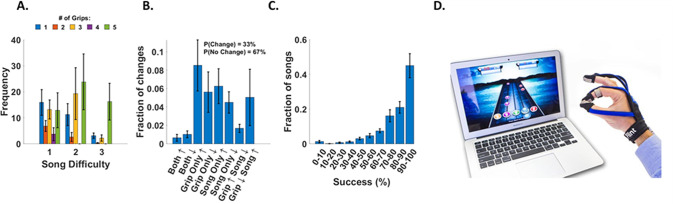
Analysis of adjustments made to game parameters across songs, as well as analysis of success at the game. **A** A bar plot of song difficulty (1: easiest, 3: hardest) versus frequency. Each bar is representative of the number of grip types used. **B** Fraction of different types of parameter changes made during gameplay. The percent of games were a parameter was not changed was 67%. **C** Fraction of songs played at different success levels. **D** MusicGlove device.

Analysis of subject game parameter settings revealed that, while subjects played the game at all three song difficulty levels, the majority of the time subjects tended to play the game at song difficulty levels 1 and 2 (Fig. 3). When subjects played the game at the easiest song difficulty setting (song difficulty = 1), they most frequently used one grip type (specifically a key-pinch grip). When subjects played at song difficulty levels 2 or 3, they tended to use all five grip types.

Regarding how subjects adjusted the game parameters, subjects made adjustments to the game on 33% of the opportunities they were given (i.e. after each song in song mode, or after each set of songs in session mode). Specifically, subjects predominately changed the number of grip types used before making any other change to the gameplay parameters (Fig. 3). Subjects also played the game at relatively high success levels, achieving a note-hitting success of greater than 82% for 66% of the 1,312 songs played (Fig. 3C). However, the note hitting success was not statistically significant compared to what was achieved in the study with sub-acute users (two sided t-test, p = 0.3). In the study with individuals in the sub-acute phase of stroke, subjects predominately played the game at song difficulty levels 1 and 2 and rarely at the most difficult level 3, which was similar to what was observed in the current study. Regarding number of grips types, subjects most frequently used 1 or 5 grip types with subjects in the current study rarely using five grip types. Parameters were changed 31% of the time, a similar rate compared to subjects in the sub-acute stroke study changing parameters. Between the two groups the level of success was also similar with subjects in the sub-acute study achieving note-hitting success of greater than 75% for 84% of the 1061 songs played. The game log data regarding challenge selection for the users in the chronic study was unavailable preventing a comparison between the present study, and that study.

**Fig. 4:**
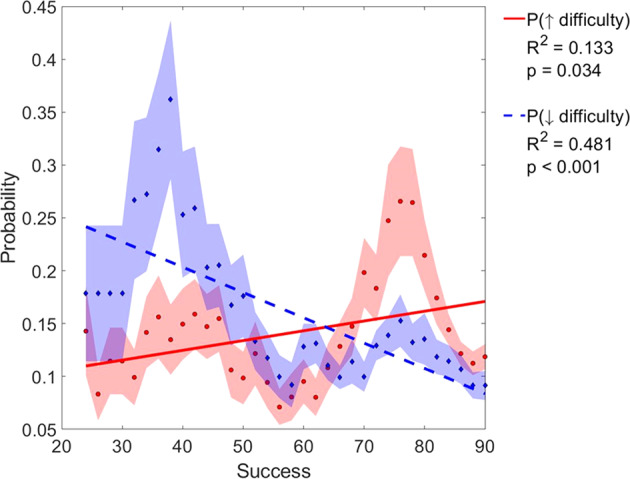
Probability of increasing (solid line) or decreasing (dashed line) game difficulty (via song difficulty or number of grips) as a function of success on previous song. Each point is the probability calculated based on all songs played within 10 points of success at that point. We required at least 100 songs to plot a point. Since subjects rarely played at low success levels, no points below 65% success were included.

Further analysis of the MusicGlove usership patterns revealed that the probability of subjects increasing the difficulty increased with success (linear regression, R^2^ = 0.13, R = 0.36, p = 0.034), and the probability of decreasing difficulty decreased with success (linear regression, R^2^ = 0.48, R = 0.70 p < 0.001) (Fig. 4). Another interesting finding was that subjects did not always modify the game in a logical manner (i.e. they did not always increase difficulty at high levels of success, or decrease difficulty at low levels of success). For example, even if subjects were able to achieve a note hitting success of 90% there still existed a finite probability of decreasing the game difficulty (10%). This same pattern of randomness was also seen at lower levels of success at the game. When the subjects had 65% level of success at the game there was an equal probability of increasing or decreasing game difficulty. We found similar results in the study conducted with individuals in the sub-acute phase of stroke. The probability of subjects increasing difficulty of gameplay also increased with success (linear regression, R2 = 0.32, p = 0.009), and the probability of decreasing difficulty decreased with success (R2 = 0.85, p < 0.001). The success level at which the probability of increasing and decreasing difficulty were equal was 74%, a number that was 9% higher than what was observed in the current study.

### Estimate of the effect of MusicGlove on hand function

**Fig. 5:**
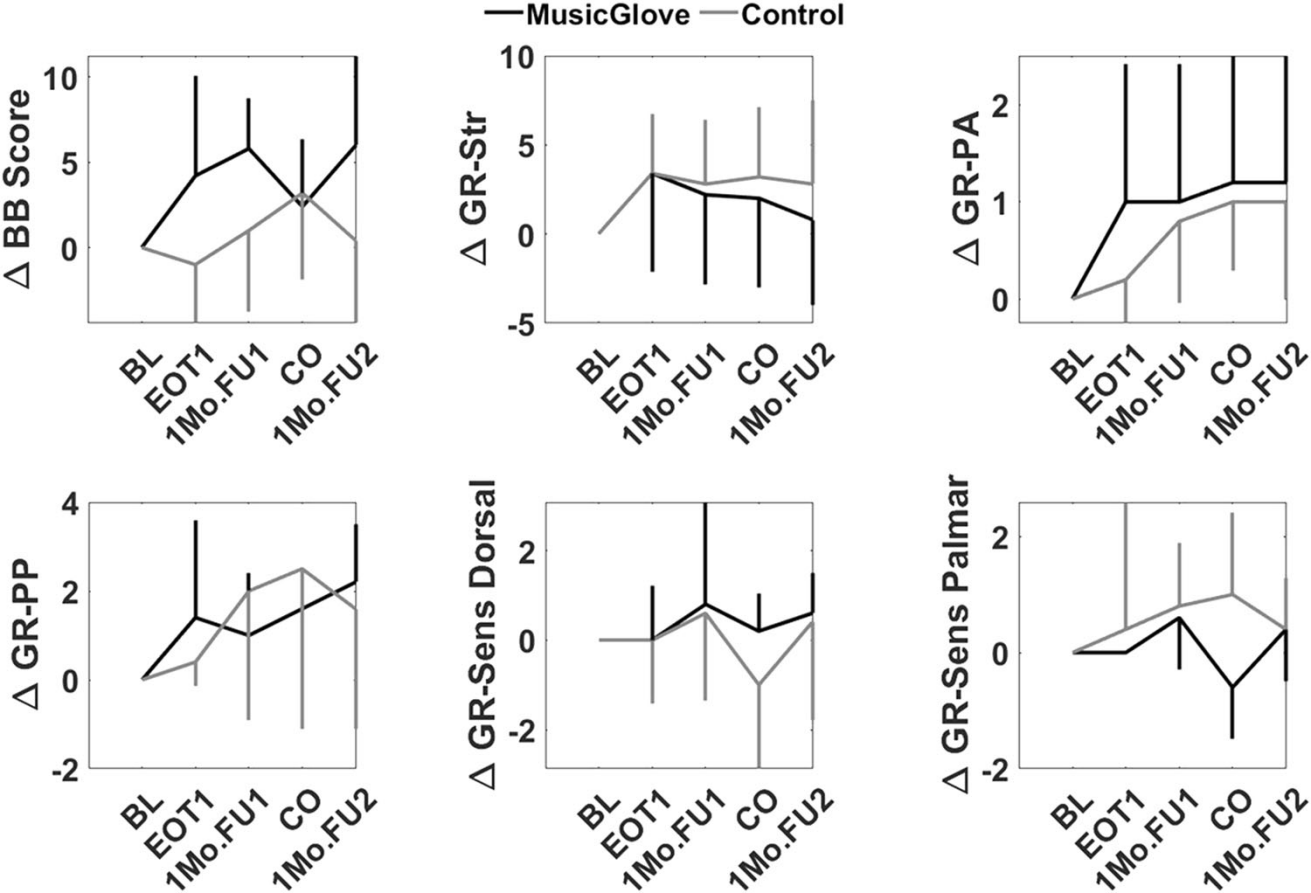
Individual trajectories of both primary and secondary outcome measures throughout the study. Vertical lines represent one SD. Additionally the average change in score was calculated relative to the baseline evaluation.

The average baseline BBT score prior to any intervention was 40.6 ± 1.7 for the MusicGlove group, and 40.0 ± 18.0 for the control group (Fig. 5). At the end of therapy after the first intervention (EOT1) the change in the BBT favored the MusicGlove group (4.2 ± 5.9 MusicGlove Group, −1.0 ± 3.4 Control Group). Examining the secondary outcome measures we observed that both the Prehension Ability (1 ± 1.4 MusicGlove, 0.2 ± 0.5 Control) and Prehension performance subtest of the GRASSP test (1.4 ± 2.2 MusicGlove, 0.4 ± 0.55 Control) favored the MusicGlove group at EOT1 (Fig. 5). The GRASSP SWMT Palmar favored the control group (0 ± 0 MusicGlove, 0.4 ± 2.2 Control). Neither the GRASSP Strength (3.4 ± 5.5 MusicGlove, 3.4 ± 3.6 Control), or GRASSP SWMT Dorsal favored either group (0 ± 1.2 MusicGlove, 0 ± 1.4 Control). No further analysis was performed on these results due to the small sample size, which we further discuss in the limitations section.

## Discussion

Spinal cord injury (SCI) is a devastating injury with injuries sustained to the spinal cord occurring at the chronic region often resulting in varying degrees of arm-hand functional loss [15]. Few studies have examined wearable grip sensing as a hand rehabilitation approach for people who have experienced a spinal cord injury (SCI). Here in this study we sought to establish the feasibility of training gripping function with MusicGlove; a wearable grip sensor at home and obtain a preliminary estimate of the therapeutic effect of MusicGlove use on hand function in subjects with chronic SCI. We also compared how users with hand impairment after SCI used the device in comparison to previously reported cohorts of stroke survivors (subacute, and chronic) who had used the device in previous studies. During the study we observed that the subjects in the present study used the device for a longer duration of time (i.e. showed increase compliance to therapy) and completed many more grips in comparison to the cohort of stroke users (sub-acute and chronic) that previously used the device. Analysis of subject game parameter settings revealed that there were some differences in challenge selection. But, generally participants in the present study modified the game parameters in a similar manner as what was seen in the previous study with stroke users, consistent with the Challenge Point Hypothesis for optimal training. Additionally, when we examined the effect of MusicGlove on hand function we observe larger increases in prehension ability, prehension performance, and BBT test scores in comparison to conventional hand therapy exercises. The constellation of these observations suggest that the

MusicGlove device is a feasible option for rehabilitation in the home setting, and potentially could help facilitate hand function, specifically unimanual gross dexterity. Below we expound upon these observations, and further discuss the potential implications for hand rehabilitation for users with chronic SCI.

### Comparison of MusicGlove usership with previous studies: amount of use

In the present study users were able to utilize the MusicGlove device to achieve a relatively higher number of grips on average (> 15,000). The number of grips that were completed were more than double the number that was completed by chronic and subacute stroke users [5, 10]. Users in this study also had higher levels of compliance than what was achieved by individuals in the sub-acute phase of stroke (6.1 h. vs. 4.1 h., respectively). Compliance to the recommended therapy time was unavailable for individuals from the chronic stroke study.

One possible reason for this difference in the number of grips completed and higher level of hourly compliance could be the level of impairment, as one would speculate that higher BBT scores at baseline indicate higher levels of hand function suggesting that completing the necessary gripping movements to play the game might be easier. However, although the BBT scores favor the SCI group it was found in all three studies that there was not a correlation between initial BBT scores and the amount of practice (# of grips completed, amount of gameplay time, rho = −0.13, p = 0.7, Spearman Correlation Coefficient). Therefore, the difference in impairment level does not adequately explain the difference in usership patterns. Another possible reason for the difference in device usership could be due to the difference in age. The average age of the users in the stroke studies (chronic, subacute) were 57 ± 10 and 62 ± 15 while for users in the SCI study the average age was 49 ± 15. It has been shown that in other related research fields of human robotinteraction and psychology, age can impact device usership. For example, older individuals tend to have more negative attitudes and are less comfortable with new technology, including computers, due to generally less experience or access, compared to the younger age groups [16]. Also it has been shown that older individuals tend to be more prone to making errors when performing computer-game based tasks [17]. However, we found no correlation between age, and device usage (rho = − 0.09, p = 0.8, Spearman Correlation Coefficient). Still there could be other complex factors such as the practical challenges of daily life that subjects face that ultimately affected device usership. Continuing to understand the factors influencing compliance is an important direction for future work and merits further exploration.

### Comparison of MusicGlove usership with previous studies: challenge selection

In a standard therapy session, a rehabilitation therapist selects the level of challenge for each subject for each therapy task based on the level of impairment of the subject. However, a common concern about self-administered care in the home setting is whether subjects will choose an appropriate level of difficulty when performing therapy tasks. This is critical as it has been suggested by the Challenge Point Hypothesis that there is an optimal level of task difficulty to promote skill development [18]. In the present study we observed that subjects made changes to the game parameters infrequently (on only 33% of songs), a number similar to that in the subacute stroke study (31%). Users in the current study predominately left the game parameters on settings that allowed them to achieve high levels of success at the game. However, the sub-acute stroke users seemed to have more consistent success at the game, as users in that study achieved note hitting success of 75% on 84% of the songs played in comparison to the current study were users achieved 82% note hitting success on 66% of the songs played. Subjects, also adjusted the game parameters in a way that was consistent with the Challenge Point Hypothesis [18–20]. In other words, subjects tended to increase the difficulty of the game if their success on the last song was high, or they would decrease the difficulty of the game if success was low.

There were a few differences in how users challenged themselves in this study compared to the sub-acute stroke study. The equilibrium point (i.e. the point at which there was an equal probability of increasing or decreasing game difficulty) occurred at a lower level of success for the current study in comparison to sub-acute study (SCI: 74%, sub-acute stroke: 65%). This indicates that individuals with SCI required a lower level of success before they attempted to try to challenge themselves more in the game, possibly because of their younger age, or higher baseline motor function (BBT scores), or fewer cognitive deficits when CNS injury occurs in the spine rather than the brain. However, despite some differences in how users challenged themselves, the common theme between both studies was that users predominately challenged themselves in a way that follows the Challenge Point Hypothesis framework, but at times still made changes to the game parameters that were counter-intuitive.

These observations have implications for how one might optimally implement rehabilitation therapy using rehabilitation technology. For example, a common strategy in many rehabilitation robots is to incorporate an adaptive algorithm to adapt task parameters to modulate the level of challenge experienced by the user after each sensed movement attempt. However, from what we have observed in the present study, adaptive algorithms that adapt parameters in a less aggressive (i.e. not adapting as often) and more stochastic (i.e. sometimes adapting in the “wrong” direction) manner may more closely resemble how individuals adapt challenge. Future studies could explore whether creating algorithms that incorporate such a strategy would lead to greater usage or better therapeutic results in comparison to algorithms that adapt task parameters after each sensed movement attempt.

### Therapeutic effect of MusicGlove on hand function in subjects with chronic SCI

For upper extremity rehabilitation after a chronic SCI conventional home based rehabilitation generally consists of a booklet of printed out hand therapy exercises. These programs as mentioned previously in the manuscript generally have low compliance. However, typically users experience a moderate benefit from those programs [21]. This makes the results of the present study surprising as at EOT users in the control group saw a decrease in BBT score and very little increase in score on the prehension ability and performance subtests of the GRASSP test. There perhaps could be a number of reasons that explain this observation. Participants could have had relatively low compliance to the therapy program, and as result experienced minimal to no gain in function. However, we cannot draw this conclusion from the present study as we did not have a way to objectively measure participant compliance to the standard exercise program, quality of the program, or the quality of the dosage. A more likely explanation could be noise due to between subject movement variability and insufficient sample size. The insufficient sample size also makes us take caution in the results observed in the experimental group, although it is promising to see that use of the MusicGlove device encouraged improvements in BBT scores, prehension ability and performance GRASSP subtests scores.

Currently, evidence-based treatment options for upper extremity rehabilitation after chronic SCI are scarce. Telerehabilitation has been identified as one possible method to deliver rehabilitation in the home setting [22–26]. In a systematic review of 12 studies in subjects with chronic SCI it was found that telerehabilitation primarily produced positive interventional impacts [25]. However, heterogeneity of studies limits the conclusive recommendations to address potential barriers to its widespread implementation. Another approach to home-based rehabilitation is the use of functional electrical stimulation (FES) systems. Many FES systems have been tested with persons with a SCI [27–34] and some studies have shown FES systems to have positive impacts on rehabilitation such as improved grasp function [28, 33, 35]. However, it still remains unclear what types of subjects benefit the most from FES-based systems as some studies report some subjects having limited benefit from the use of such systems. There has also been an increase in the development of hand exoskeletons [36–43], but few have translated to home testing. Further, In the chronic phase rehabilitation strategies often focus on compensation. This work supports the notion that individuals with incomplete SCI can improve hand motor function through high repetition practice of sensed gripping movements via a wearable grip sensor at home although future studies with larger sample size will be necessary to validate improvements observed in this study.

### Limitations

Budgetary constraints hindered our ability to recruit the planned number of subjects for the study (N = 20 for each group), and therefore, definitive conclusions should not be made on the therapeutic effect until a study with a larger number of subjects can be performed. Additionally, 80% of the participants in the study were male, and one individual in the study would not be considered “chronic” based on the WHO definition. Both of these participant characteristics limit the generalizability of the results. There was also a large difference in the amount of time passed since incidence of SCI between the two groups. Specifically, the experimental group had less time passed since the incidence of SCI compared to the control group. This potentially could have influenced the expected therapeutic benefit but we think this is unlikely given that spontaneous recovery has at this point plateaued. Subject compliance was low in terms of logging how many hours conventional hand therapy training was performed. This prevented us from making comparisons on program compliance between the two different interventions or analyzing possible dose effects of the conventional hand therapy training approach. Despite these limitations this data does provide an initial estimate of the effect size, which in the future can be used for planning larger studies.

## Conclusions

From this study we have shown that MusicGlove (a wearable grip sensor) is a feasible option for home based grip training for individuals with chronic SCI. On average subjects performed at least 6 hours of MusicGlove usage over the course of the three-week study while completing approximately 16,000 grips. This number of grips was nearly twice as many gripping movements in comparison to individuals from the sub-acute and chronic stroke populations, and is far greater than the number of movements typically achieved during traditional rehabilitation [44]. This would suggest that the device was engaging to users and encouraged a high level of compliance to the training. Further, subjects played the game in a similar manner to that seen with subacute stroke survivors—i.e. playing mostly at high success levels, infrequently making parameter changes to the game, and roughly following what would be suggested by the Challenge Point Hypothesis when a change was made. Finally, it appears this training approach could help facilitate functional hand recovery given the improvements in the Box and Blocks test scores, and prehension ability as measured by the GRASSP test at the end of therapy after the first intervention.

However, at this point, it is still unclear if this ultimately translates into long-term improvement in hand function with individuals with chronic spinal cord injuries. Future studies will need to examine the exact timing and hand function parameter threshold that are optimal for individuals with SCI in a larger cohort of subjects in order to better understand the impact of such sensor-based rehabilitation therapies that target the hand after SCI.

## Supplementary information

Supplementary Information

## Data Availability

The datasets generated and analyzed during the current study are available from the corresponding author upon reasonable request.
