# Supplementary material for: Feasibility of home hand rehabilitation using musicglove after chronic spinal cord injury
Source: Spinal Cord Ser Cases. 2022 Nov 9;8:86. doi: 10.1038/s41394-022-00552-4 (PMC9643482; doi:10.1038/s41394-022-00552-4)
Supplement: Supplementary file 1 — Supplementary Information [file 41394_2022_552_MOESM1_ESM.pdf]

Total 18

COMMENTS:

Please complete the following exercises 3 times a week for an hour each time.

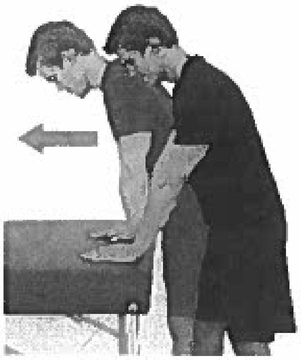

WRIST EXTENSION STRETCH - TABLE

Place boths hand on a table as shown and gently lean forward until a stretch is felt.

Repeat	3 Times
Hold	10 Seconds
Complete	2 Sets
Perform	2 Time(s) a Day

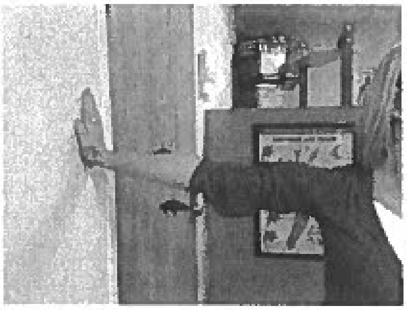

Wrist Flexor Stretch

Gently pull wrist into extension with elbow extended, while keeping shoulder down.

Repeat	4 Times
Hold	10 Seconds
Complete	2 Sets
Perform	2 Time(s) a Day

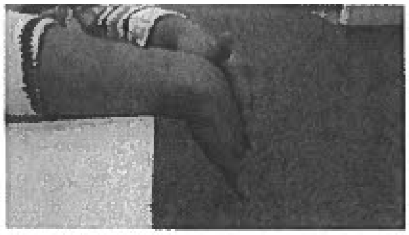

prom wrist flexion

rest your forearm on the edge of a table, palm facing down. Now use your other hand to bend your wrist down towards the floor until you feel a stretch. Hold for 30 seconds, repeat 5 times, 3 times a day.

Repeat	10 Times
Hold	5 Seconds
Complete	2 Sets
Perform	2 Time(s) a Day

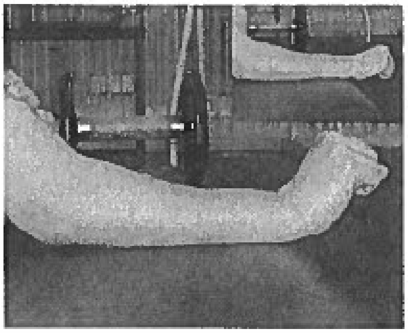

Wrist Extension

Start with wrist at edge of table, palm facing down. With wrist slightly off the edge of the table, curl wrist up and back down.

Repeat	10 Times
Hold	5 Seconds
Complete	2 Sets
Perform	2 Time(s) a Day

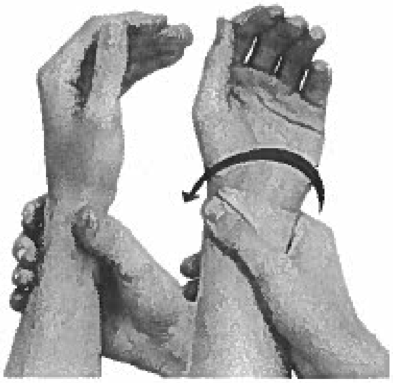

WRIST SUPINATION STRETCH

Grasp your wrist as shown and gently turn your affected wrist towards palm face up.

Keep your elbow bent and by the side of your body.

Repeat	10 Times
Hold	5 Seconds
Complete	2 Sets
Perform	2 Time(s) a Day

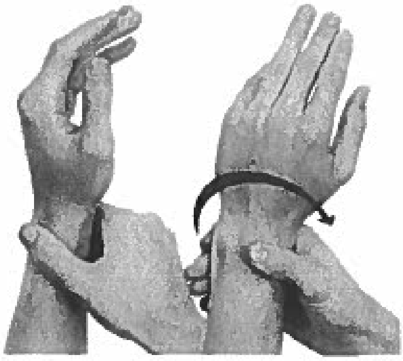

WRIST PRONATION STRETCH

Grasp your wrist as shown and gently turn your affected wrist towards palm face down.

Keep your elbow bent and by the side of your body.

Repeat	10 Times
Hold	5 Seconds
Complete	2 Sets
Perform	2 Time(s) a Day

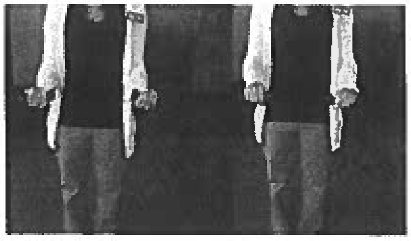

Pronation/Supination

With elbow bent 90 degrees, turn palm up and palm down alternately.

Repeat	10 Times
Hold	10 Seconds
Complete	2 Sets
Perform	2 Time(s) a Day

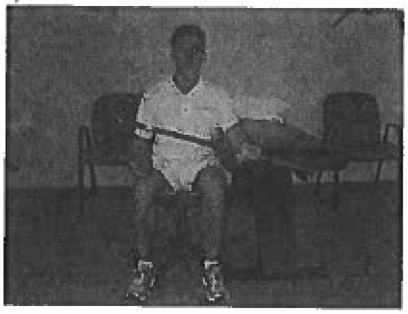

Cane Supination Pronation

Hold cane with hand over edge of table, elbow bent to 90 deg.
Rotate cane palm up, palm down as far as possible.

Repeat	10 Times
Hold	5 Seconds
Complete	2 Sets
Perform	2 Time(s) a Day

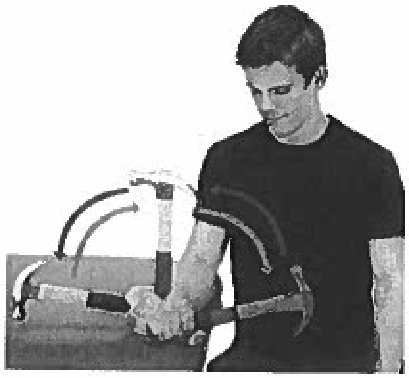

HAMMER PRONATION SUPINATION

Slowly lower a hammer towards the inside and then outside of the body as shown.

Repeat	10 Times
Hold	5 Seconds
Complete	2 Sets
Perform	5 Time(s) a Day

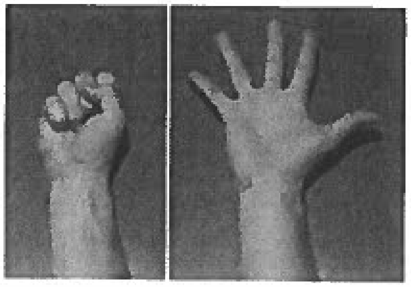

Finger Abduction

Start with a fist
Then spread fingers out as far as they will go
Repeat

Repeat	10 Times
Hold	5 Seconds
Complete	2 Sets
Perform	2 Time(s) a Day

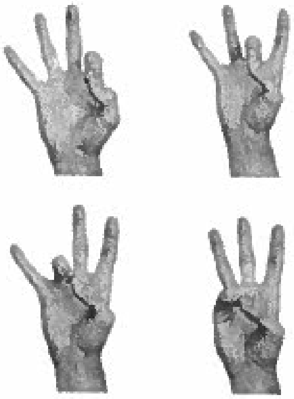

FINGER OPPOSITION COMBO

Start with an open palm and fingers extened.

Next, touch the tips of the first and second fingers. Then return to open palm.

Next, touch the tips of the first and third fingers, etc until all fingers have performed as shown.

Repeat	10 Times
Hold	3 Seconds
Complete	2 Sets
Perform	2 Time(s) a Day

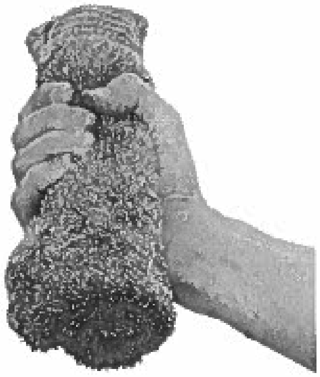

TOWEL GRIP

Place a rolled up towel in your hand and squeeze.

Repeat	10 Times
Hold	5 Seconds
Complete	2 Sets
Perform	1 Time(s) a Day

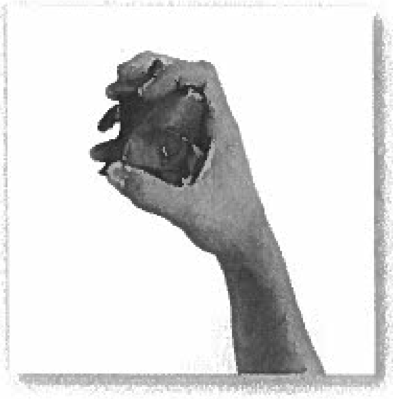

PAPER CRUMPLE

Take a piece of paper and crumple it.

Repeat	10 Times
Hold	4 Seconds
Complete	1 Set
Perform	1 Time(s) a Day

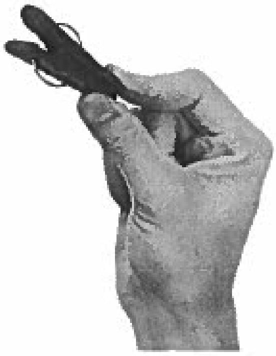

CLOTHES PIN PINCH

Use 2 fingers and pinch a clothes pin or chip clip.

Repeat	10 Times
Hold	1 Second
Complete	2 Sets
Perform	2 Time(s) a Day

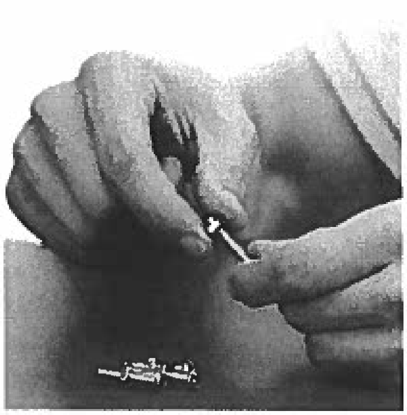

SCREW AND THREAD NUT

Hold a screw with one hand and thread a nut on the screw with the target hand.

Repeat	10 Times
Hold	1 Second
Complete	2 Sets
Perform	1 Time(s) a Day

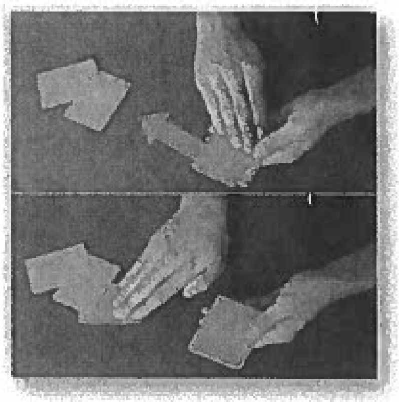

CARD SLIDE

Hold a deck of cards with your unaffected hand and slide one card at a time to the side with your affected hand.

Repeat	12 Times
Hold	1 Second
Complete	2 Sets
Perform	2 Time(s) a Day

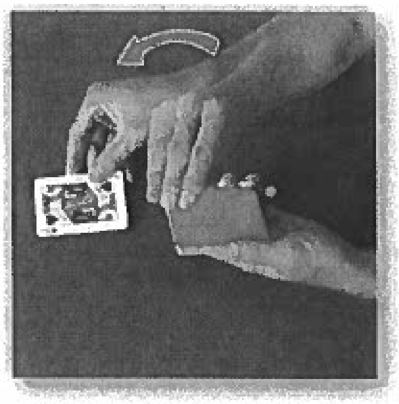

CARD FLIP

Hold a deck of cards with your unaffected hand and flip one card at a time with your affected hand.

Repeat	12 Times
Hold	1 Second
Complete	2 Sets
Perform	2 Time(s) a Day

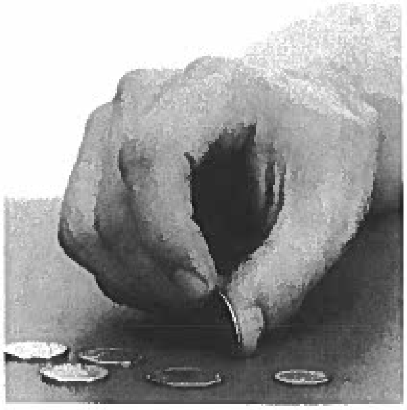

COIN FLIP

Place various coins on a table and flip each coin with your affected hand.

Repeat	12 Times
Hold	3 Seconds
Complete	2 Sets
Perform	2 Time(s) a Day
